# Age-stratified multimodal MRI and machine learning to explore autism-related brain characteristics in youth

**DOI:** 10.3389/fpsyt.2026.1841698

**Published:** 2026-07-02

**Authors:** Garazi Casillas Martinez, Anthony Winder, Kimberly Amador, Eneko Uruñuela, Matthias Wilms, Sarah J. MacEachern, Nils D. Forkert

**Affiliations:** 1 Department of Radiology, University of Calgary, Calgary, AB, Canada; 2 Hotchkiss Brain Institute, University of Calgary, Calgary, AB, Canada; 3 Alberta Children’s Hospital Research Institute, University of Calgary, Calgary, AB, Canada; 4 Department of Clinical Neurosciences, University of Calgary, Calgary, AB, Canada; 5 Department of Radiology, University of Michigan, Michigan, MI, United States; 6 Department of Pediatrics, University of Calgary, Calgary, AB, Canada

**Keywords:** autism, development, diffusion MRI, machine learning, neuroimaging, resting-state functional MRI, structural MRI

## Abstract

**Purpose:**

Autism is a common neurodevelopmental condition (NDC) that is characterized by restricted, repetitive behaviors and social communication differences that can impact the daily functioning of individuals. The clinical diagnosis of autism can be challenging, mainly due to its behavioral variability and frequent co-occurrence with other NDCs. This study investigates the ability of machine learning-based classification models trained using multimodal neuroimaging data combined with feature-importance analyses to identify development-specific brain characteristics associated with autism.

**Approach:**

A total of 144 participants aged 5 to 18 years with structural MRI (sMRI), diffusion MRI (dMRI), and resting-state functional MRI (rs-fMRI) data available were obtained from the Autism Brain Imaging Data Exchange (ABIDE) database. Radiomic features were extracted from each MRI data modality and used to train support vector machine (SVM) classifiers to identify neuroimaging patterns associated with autism. Single MRI modality classifiers, as well as one combining all three modalities, were trained for comparison purposes. To investigate age-specific effects, the same approach was followed for three age sub-groups: younger children (5–11 years), adolescents (12–18 years), and the entire 5–18 years age cohort. Model performance was evaluated using leave-one-out cross-validation across 30 diagnosis-balanced data splits. Feature-importance analyses were conducted to identify the most important neuroimaging features for classification.

**Results:**

The classification accuracies of the unimodal models ranged from 68.3% to 75.3% for sMRI, from 69.3% to 77.6% for dMRI, and from 66.3% to 69.9% for rs-fMRI data across age groups. Among all single imaging modalities and age groups, dMRI showed the highest performance with a 77.6% accuracy in younger children (5–11 years). The multimodal approach improved classification performance when compared to the unimodal models in all age groups, achieving accuracies of 78.9%, 76.7%, and 70.5% in the younger, adolescent, and entire age cohorts, respectively. Our findings indicate that multimodal classifiers integrating complementary structural, microstructural, and functional imaging features result in a more comprehensive representation of brain features that strengthens model performance. The most informative brain regions for classification differed between children and adolescents while several diffusion-derived features significantly correlated with social responsiveness scores, emphasizing the clinical importance of studying white and gray matter microstructure in autism.

**Conclusions:**

This study demonstrates the potential of multimodal neuroimaging-based machine learning models to identify development-specific biomarkers associated with autism. The results highlight the value of integrating age-stratified analyses of multimodal neuroimaging to better capture autism-associated developmental brain characteristics. The framework adopted in this study could be extended to explore other NDCs in the future.

## 1 Introduction

Autism is a complex neurodevelopmental condition (NDC) related to brain development that can involve several differences compared to non-autistic individuals with respect to social communication, restricted interests, and repetitive behaviors Hodges et al. (1). Approximately 1% of the worldwide population is autistic Zeidan et al. (2). Symptoms often appear early in childhood and can have an impact on the daily functioning of individuals, families, and caregivers Pandina et al. (3). The current clinical diagnostic process is primarily based on behavioral questionnaires and clinical observations conducted by trained healthcare professionals. Depending on where the child is located, assessments may be performed by pediatricians or developmental pediatricians, often in collaboration with speech-language therapists and psychologists. However, the process is often time-consuming Nomi and Uddin (4) and resource intensive Kamp-Becker et al. (5), since many professionals with extensive training and background knowledge need to be involved Kamp-Becker et al. (5). Moreover, some diagnostic tools utilized can be subjective Eslami et al. (6), and can vary depending on the clinician’s previous experience and the diagnostic criteria. Further complicating the diagnostic process is that autism often co-occurs with other neurodevelopmental and psychiatric conditions Romero et al. (7) Khachadourian et al. (8), with which there may be a significant symptom and behavioral overlap Craig et al. (9). Despite extensive research, there is still no universal, well-accepted, quantitative biomarker of autism for clinical diagnosis, which reflects the complexity and variability of the condition Herbert (10).

Over the past two decades, advances in neuroimaging techniques have been instrumental to study the potential biological mechanisms underlying autism Halliday et al. (11). Using these techniques, atypical patterns of brain development, structure, and function have been observed in autistic individuals Rashid et al. (12). Within this context, magnetic resonance imaging (MRI) is among the most commonly utilized neuroimaging techniques in autism research, with structural MRI (sMRI), diffusion MRI (dMRI), and resting-state functional MRI (rs-fMRI) being some of the most studied MRI sequences. Specifically, sMRI provides quantitative measures of the volume and shape of brain regions, and it is used to identify morphological differences between individuals or groups. Using sMRI data, previous studies have observed several structural brain differences in autistic individuals when compared to typically developing individuals. For example, volumetric differences and cortical thickness alterations have been observed in multiple brain regions in autistic toddlers, children, and adolescents compared to their respective typically developing peers (*e.g.*^13^–^32^). Importantly, previous meta-analyses Stanfield et al. (18)Amaral et al. (33) have reported that some changes (*e.g.*, enlargement of the amygdala) are specific to age, suggesting that age should be considered a key factor when studying structural brain differences in autism Anagnostou and Taylor (34).

Diffusion MRI measures the diffusion of water molecules in the brain, allowing researchers to noninvasively study white matter (WM) and gray matter (GM) tissue microstructure. Mean diffusivity (MD) and fractional anisotropy (FA) are two commonly studied diffusion metrics that can be computed from diffusion tensor imaging (DTI). Briefly described, MD captures the overall magnitude of water diffusion, while FA informs about how organized or aligned the tissue structure is. Previous neuroimaging work has reported decreased FA and increased MD values across multiple WM tracts in autistic children and adolescents when compared to typically developing individuals (35–41). However, other studies have observed the opposite pattern of elevated FA in pre-school aged children under the age of five Shen et al. (42) Andrews et al. (43). These findings highlight the heterogeneity of brain microstructural changes in autistic individuals across development. Overall, age-related differences in DTI-derived metrics in autistic individuals have been less studied in cortical GM compared to WM tissue DiPiero et al. (44).

Resting state-functional MRI (rs-fMRI) measures spontaneous brain activity fluctuations when an individual is at rest, enabling to study the brain’s intrinsic neural dynamics underlying cognitive functions Buckner et al. (45). Within this context, functional connectivity (FC) is a commonly studied rs-fMRI-derived metric and refers to the temporal correlation of the rs-fMRI-derived signal between spatially distant brain regions Biswal et al. (46). Despite extensive research, rs-fMRI findings in pediatric autism remain inconsistent across development, with prior work reporting hyper-connectivity (reduced connectivity between brain regions) during childhood and a shift toward hypo-connectivity (increased connectivity) during adolescence Uddin et al. (47). Beyond brain region-wise connectivity analyses, using graph theoretical approaches, researchers can explore how information is integrated, centralized, and segregated across the entire brain network. Importantly, using graph-theoretical analyses, differences in brain network organization have been previously observed between autistic and typically developing children, adolescents, and young adults Rudie et al. (48) Keown et al. (49) Balardin et al. (50) Zeng et al. (51) Ray et al. (52).

Overall, previous neuroimaging findings in autism research remain largely inconsistent, partially because many studies include participants across broad age ranges, complicating the identification of development-specific brain characteristics Halliday et al. (11). Since neuroimaging data analyses have considerably advanced our understanding of the autistic brain through group-wise analyses, applying artificial intelligence (AI) methods MacEachern and Forkert (53) Lo Vercio et al. (54) to this data has become an important direction to move forward in autism research. This AI research has been made possible by the growing availability of large and public neuroimaging data repositories. For example, the Autism Brain Imaging Data Exchange (ABIDE) Di Martino et al. (55, 56 is one of the largest, publicly available databases containing neuroimaging information from autistic and typically developing individuals. As a result, machine learning (ML) models utilizing this database have been widely used to investigate neuroimaging patterns that characterize autistic and typically developing individuals. However, ML studies combining all three modalities (sMRI, rs-fMRI and DTI) remain scarce. Such a multimodal integration could provide complementary information about brain morphology, microstructure, and function that unimodal or bimodal approaches may not fully capture. Importantly, the potential value of integrating these three modalities into age-stratified diagnostic models that specifically focus on young autistic populations has not yet been examined. The few ML studies that have combined fMRI, sMRI, and DTI data to date have either relied on participant cohorts spanning wide pediatric age ranges (*e.g.*, 5 to 18 years He et al. (57)) or even extended their analyses to include adult populations Varshney et al. (58). Since the brain undergoes significant changes across development, combining data across large age cohorts when studying autism can lead to confounding results O’Hearn and Lynn (59). Moreover, given the importance of early intervention Okoye et al. (60), and growing evidence suggesting that autism-related brain differences are highly age-dependent (4, 18, 33, 47, 61–71), it is important for ML models to target narrow pediatric age cohorts. This approach may reduce developmental heterogeneity and facilitate a more clear identification of autism-associated brain characteristics across development. Finally, neuroimaging-based ML studies in autism research are often considered black box models that provide limited exploration into the brain features contributing to classification decisions, which can hinder validation and trust among clinicians. Thus, incorporating feature importance methods into ML predictive models of autism can help address this limitation, as well as identify and quantify new biomarkers of autism.

This study aims to address the mentioned gaps by evaluating neuroimaging-based classification frameworks for autism within narrowly defined developmental stages. To do so, we train and test ML models in three pediatric cohorts: children aged 5–11 years, adolescents aged 12–18 years, and the combined 5–18 age group, and we examine:

1. How model performance differs across narrow pediatric age groups compared with the broader 5–18 cohort, and how age stratification influences both classification performance and feature-importance.
2. Whether multimodal models integrating sMRI, dMRI, and rs-fMRI outperform unimodal models trained on a single MRI data modality, and how these performance patterns vary across pediatric age groups.
3. Which MRI-derived features contribute most to classification within each age group. Additionally, we explore whether specific brain regions overlap across MRI data modalities and if this overlap varies across age groups, to identify if there are any neuroanatomical biomarkers carrying strong predictive information.

## 2 Materials and methods

The data for this study were obtained from ABIDE II Di Martino et al. (56), which includes data from autistic and typically developing individuals aged 5 to 64 years. Briefly described, the ABIDE database is an open-access resource containing only fully anonymized data. All sites contributing data to ABIDE obtained informed consent and local Institutional Review Board (IRB) approval for initial data collection and sharing of the de-identified data. As our secondary study only used anonymized data from ABIDE, no additional ethical approval was required.

The inclusion criteria for this secondary study included pediatric participants aged 5 to 18 years with sMRI, dMRI, and rs-fMRI data available. After image preprocessing, the final dataset consisted of 144 participants (88 autistic and 56 non-autistic) from four different data acquisition sites within the ABIDE II database, these being New York University (NYU), San Diego State University (SDSU), Trinity College Dublin (TCD), and Barrow Neurological Institute (BNI). Additional demographic information about participants is shown in **Supplementary Tables 1**–**3**.

### 2.1 Age stratification rationale

Based on the entire 5–18 age cohort used in this study, participants were further divided into two narrow developmental age groups for ML model training and testing: younger children aged 5–11 and adolescents aged 12–18 years. The motivation for dividing the dataset into these age windows was two-fold. First, the 5–11 age window approximates late childhood or pre-adolescence, while from around the age of 12 onward, many individuals are entering or already in puberty, a developmental period in which children experience physical and hormonal changes to reach sexual maturity Cleveland. Puberty has been strongly associated with brain maturation Dai and Scherf (72) [*e.g.*, accelerated puberty has been linked with accelerated brain development Holm et al. (73)]. Independent of puberty-related processes, brain maturation is dynamic during childhood and adolescence Forkert et al. (74). For example, adolescence (which roughly ranges from 12 to 18 years of age), represents the developmental window with most accelerated cortical thinning Zhou et al. (75). Therefore, utilizing the 11–12 years as a boundary was considered meaningful from a developmental perspective as it separates the pre-pubertal or pre-adolescence group from a group that is transitioning toward adolescence, a period in which multiple brain developmental changes start to emerge.

### 2.2 Image preprocessing

T1-weighted images were selected as the source of sMRI data, as they provide good anatomical detail at high spatial resolution. The selected T1-weighted MRI scans underwent typical preprocessing. First, N4 bias field correction was applied to correct image intensity inhomogeneities using the N4ITK bias field correction method implemented in Advanced Normalization Tools (ANTs) Avants et al. (76). Next, skull stripping was performed to remove non-brain tissues using Hd-BET Isensee et al. (77). Affine registration of each T1-weighted MRI scan to the NIHPD 7.5-13.5-year-old asymmetric atlas Fonov et al. (78) was then applied for spatial normalization using ANTs. Next, due to global intensity variations caused by differences in MRI acquisition protocols, histogram matching was performed to standardize image intensity values using SimpleITK Yaniv et al. (79), ensuring that images had a consistent intensity distribution across participants. The NIHPD 7.5-13.5-year-old asymmetric atlas was used as the reference image for the histogram matching process. The preprocessed images were center cropped from their original size of 197×233×189 voxels to an extent of 161×201×165 voxels, which was large enough to fully capture the brain in all participants.

Diffusion MRI data was preprocessed using the FMRIB Software Library (FSL, version 6.0) Smith et al. (80). First, eddy current and motion correction was applied to correct artifacts and misalignments introduced by head motion or changes in the direction of the gradient field of the MRI scanners, using the non-diffusion-weighted (*b* = 0) image as a reference. Subsequently, brain extraction was performed using the Brain Extraction Tool (BET) Smith (81) to generate binary brain masks and remove non-brain tissue. Next, FA and MD maps were generated for each participant using the *dtifit* tool from FSL, based on least squares estimation.

**Figure 1:**
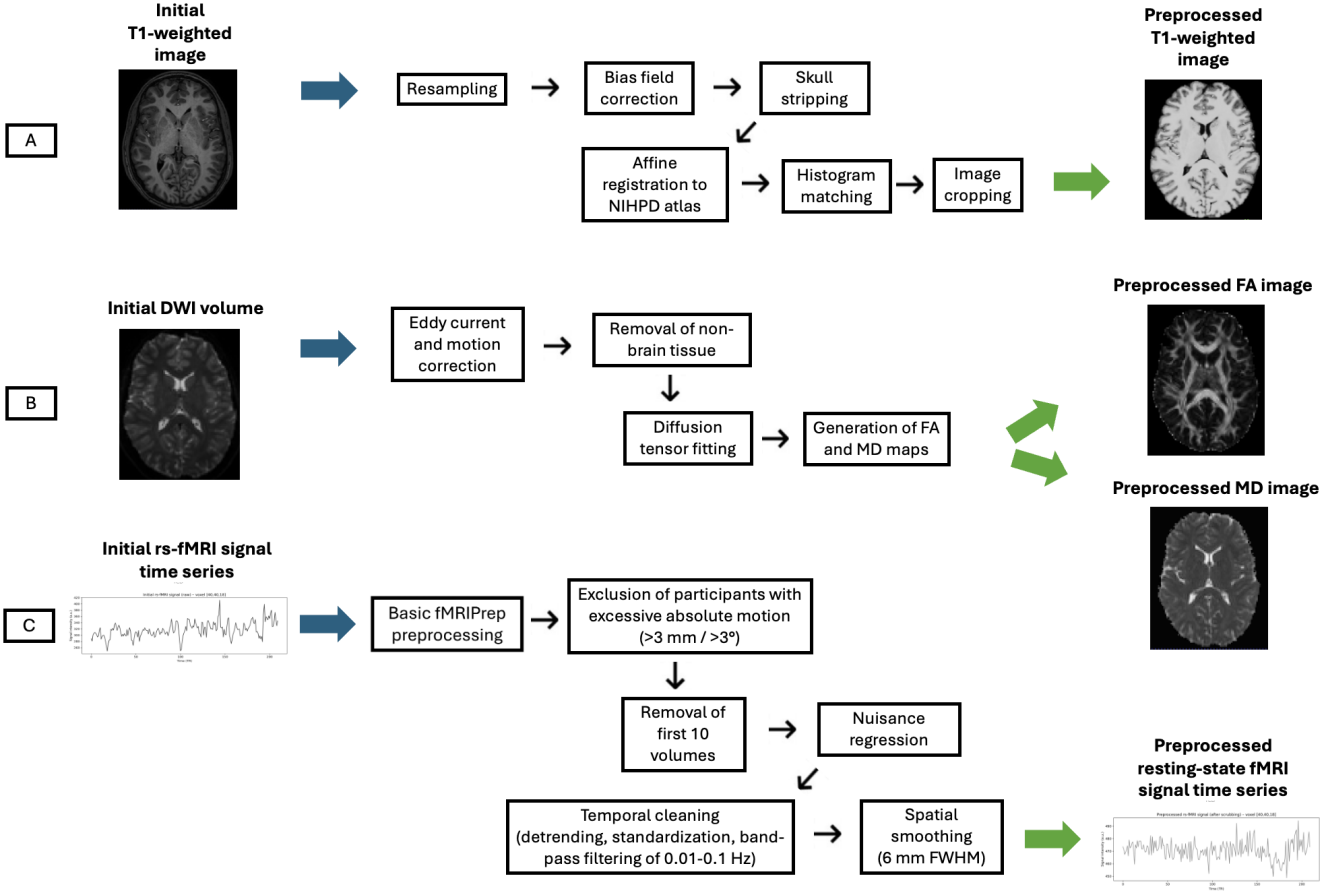
Preprocessing pipeline for sMRI **(A)**, dMRI **(B)**, and rs-fMRI **(C)** data.

Resting-state fMRI data was minimally preprocessed using the fMRIPrep pipeline Esteban et al. (82). This pipeline included anatomical-functional co-registration using a T1 weighted-based initialization, susceptibility-distortion correction, and normalization to MNI152NLin6Asym space at 2 mm isotropic resolution. This primary preprocessing step generated preprocessed blood-oxygen-level–dependent (BOLD) images in standard space along with the corresponding confound regressors. Subsequent preprocessing steps were performed using custom Python scripts. Briefly described, subjects exhibiting excessive absolute head motion (*>* 3 mm translation or *>* 3^°^ rotation) were excluded Power et al. (83), and the first 10 volumes were discarded. The time series were then cleaned by regressing out nuisance regressors comprising six rigid-body motion parameters (three translations and three rotations), alongside physiological white matter and cerebrospinal fluid signals, detrended, standardized, temporally band-pass filtered (0.01–0.1 Hz), and spatially smoothed applying a 6 mm full-width at half-maximum (FWHM) Gaussian kernel using the Nilearn library Abraham et al. (84). **Figure 1** illustrates the preprocessing pipeline for sMRI, dMRI, and rs‑fMRI.

### 2.3 Feature extraction

Once the neuroimaging data was preprocessed, radiomic features were extracted from each neuroimaging data modality.

T1-weighted MRI scans. Regional GM brain volumes were calculated for each of the 90 cortical and subcortical brain regions of interest (ROIs) from the Automated Anatomical Labelling (AAL) atlas Tzourio-Mazoyer et al. (85). Similarly, regional WM volumes were calculated from the JHU-ICBM-DTI-48 WM atlas Hua et al. (86), which comprises 48 WM tract labels. For GM volumes, to obtain brain parcellations specific to each participant, the Collin T1 template Collins et al. (87) was skull-stripped by applying a pre-computed binary brain mask using SimpleITK Yaniv et al. (79) and non-linearly registered to each participant’s native T1 space using NiftyReg Modat et al. (88). More precisely, a rigid alignment was initially performed, followed by a non-linear variant of the free-form deformation algorithm Rueckert et al. (89), which resulted in a deformation field mapping the template space to each participant’s anatomical T1-weighted MRI space. Subsequently, this deformation field was applied to the AAL-90 parcellation atlas, while resampling the brain regional labels from the atlas into each participant’s anatomical T1-weighted MRI space. This step was performed using nearest-neighbor interpolation to preserve the discrete labels of brain regions. The resulting AAL-90 parcellation in participant-space was afterwards used to calculate regional GM volumes, by superimposing it to each anatomical T1-weighted MRI scan. For WM regions, the same procedure was followed. In this case, the reference T1 template in atlas space used was the masked ICBM AVG 152 T1 Mazziotta et al. (90). After computing the registrations described above and obtaining the JHU-ICBM-DTI-48 WM parcellations in each participant’s native space, regional WM volumes were calculated. Both, GM and WM volumes, were afterwards normalized by the total volume of their respective parcellation atlases, to ensure comparability across participants, following the proportion approach described in previous work O’Brien et al. (91).

Diffusion MRI scans. Median diffusion values were calculated from the computed DTI-derived FA and MD maps for each GM brain region defined in the AAL-90 atlas and from each WM region defined in the JHU-ICBM-DTI-48 WM atlas. First, the DTI-derived FA and MD maps were aligned to each participant’s anatomical T1-weighted MRI space. To do so, a rigid registration of each (*b* = 0) image to each participant’s T1-weighted MRI scan was performed using NiftyReg Modat et al. (88), and the resulting transformation was subsequently applied to both the FA and MD maps, leading to diffusion metrics in participant-specific space. The AAL-90 and JHU-ICBM-DTI-48 WM atlases in each participant’s T1-weighted MRI space were then superimposed on top of the FA and MD maps and used to calculate median diffusion values for each GM and WM brain region. Median instead of average values were used to better account for partial volume effects. More precisely, partial volume effects can inflate FA and MD values in certain brain regions, such as the thalamus, due to its proximity to the ventricles, which have high MD values due to the freely moving water. This overlap can result in higher-than-expected diffusion values in some brain regions. To further prevent this potential issue, a 2×2×2 voxels erosion was applied to each brain atlas region before calculating the median diffusion values.

**Figure 2:**
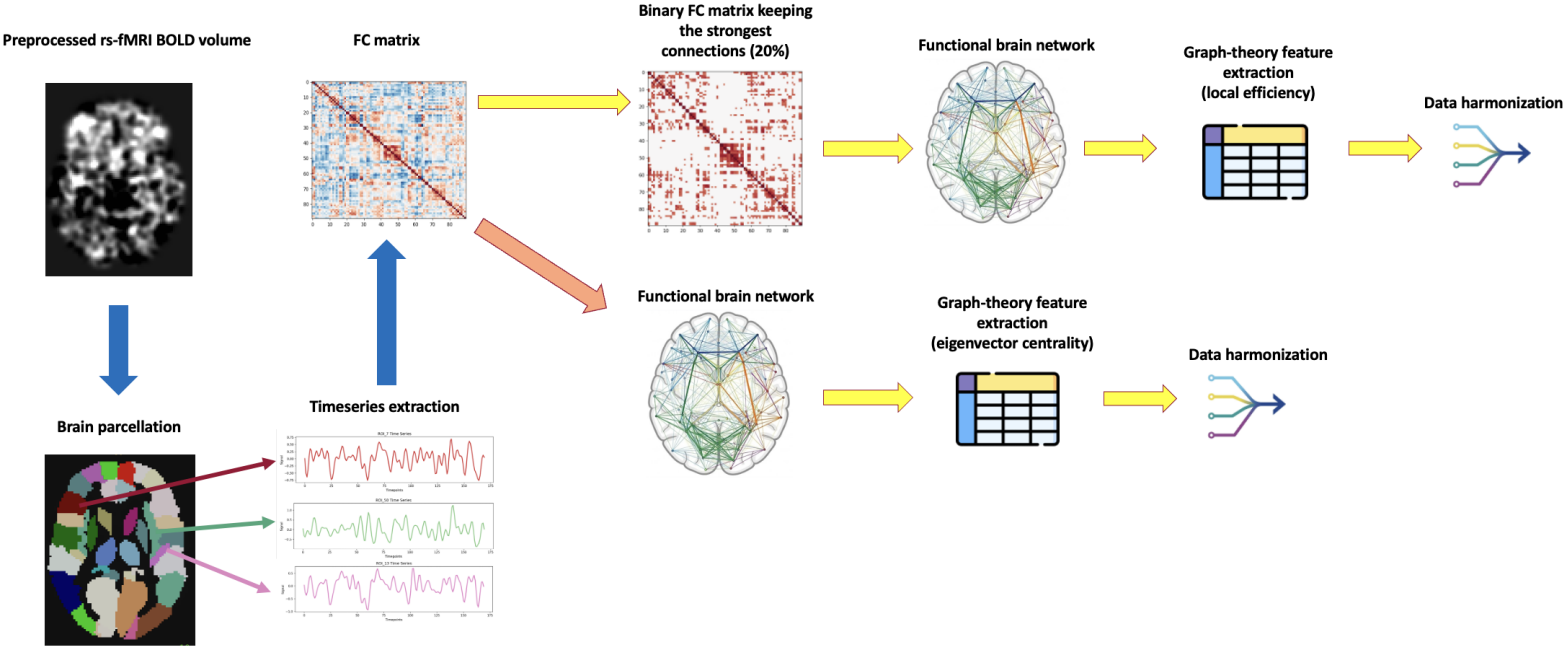
Pipeline for calculating graph-theory–based features. The functional brain network and data harmonization icons were created with Gemini.

Due to differences in scanners from the different data acquisition sites from the ABIDE II database, the structural and diffusion data was harmonized across sites using the ComBat data harmonization tool Fortin et al. (92), controlling for sex, age, and diagnostic group (autistic or non-autistic). Therefore, each participant’s sMRI data was represented by 138 features (90 GM and 48 WM volumes), while dMRI data was represented by 276 features (180 median FA and MD values from GM, and 96 median FA and MD values from WM regions). **Figure 2** provides a detailed visualization of the sMRI and dMRI feature extraction process for GM (please note that the same procedure was followed to calculate features from WM regions, using the respective WM atlas).

**Figure 3:**
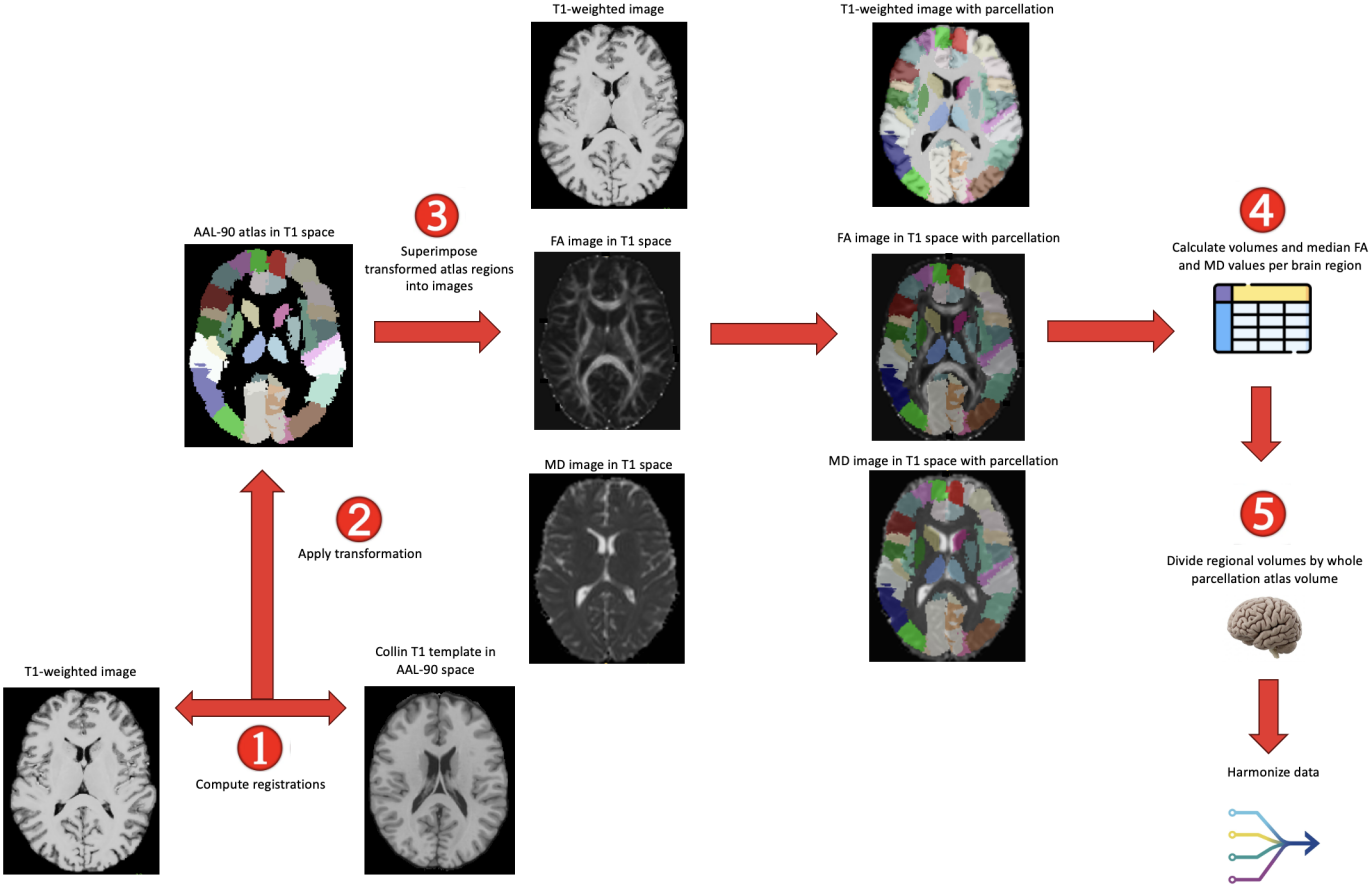
Pipeline for calculating the mean volumes, median FA, and median MD values for each GM brain region from the AAL-90 atlas. Icons created with Gemini.

Resting-state fMRI scans. The AAL-90 atlas was used to extract the mean rs-fMRI time series for each of the 90 ROIs. To do so, the atlas was first registered to the same MNI space of the preprocessed BOLD images to accurately align the parcellations with the rs-fMRI BOLD scans. Functional connectivity matrices were then computed by calculating Pearson correlations between each pair of average time series across the AAL-90 regions. Next, graph-theory based features were calculated for each brain region. More precisely, a centrality measure (eigenvector centrality) and a segregation measure (local efficiency) were calculated. These features were computed using the network library in Python Hagberg et al. (93). Briefly described, eigenvector centrality measures the influence that a node (*e.g.*, a brain region) has in the whole network, and assesses if that node is connected to other central nodes. This measurement was calculated on a weighted graph, which preserves the strength of the functional connections between brain regions, with edge weights given by the absolute Pearson correlation values to preserve the relevance of negative connections. In contrast, local efficiency measures the level of efficiency of the information exchange among the neighbors of a node in the network. Briefly described, efficiency refers to how easily the information can flow between nodes. This feature was calculated on a binary graph that was thresholded at 20% edge density to preserve the strongest connections. The thresholding step facilitates the calculation of the local efficiency metric that depends on network topology, while also reducing the computational burden of analyzing the graph Garrison et al. (94), and limiting the influence of weak or noisy connections. Due to potential differences across scanners and data acquisition protocols, graph-theory features were also harmonized across imaging sites using Combat Fortin et al. (92), controlling for sex, age, and diagnostic group. As a result, the rs-fMRI data for each participant was represented as a vector of 180 features (90 eigenvector centrality features and 90 local efficiency features). These features were only computed using the AAL-90 atlas for GM regions, since studying function in WM remains controversial Gawryluk et al. (95). **Figure 3** provides a detailed diagram of the rs-fMRI preprocessing pipeline described above.

In summary, after pre-processing each participant’s neuroimaging data was represented by 138 sMRI-derived features, 276 dMRI-derived features, and 180 rs-fMRI-derived features.

### 2.4 Model design, training, and evaluation

The neuroimaging feature set was used to train multiple ML models for binary autism classification, separately for the 5–11, 12–18, and 5–18 age cohorts. Analyses were conducted using both unimodal and multimodal models. The unimodal ML models were trained separately using features derived from one neuroimaging data modality at a time, with age and sex included as additional demographic features, enabling to assess the discriminative power of each modality independently. To investigate the potential benefit of multimodal data integration, radiomic features from all three neuroimaging modalities were concatenated and combined with the age and sex variables to train the multimodal ML models. Before model training, all features (including radiomic features, age, and sex) were z-score normalized separately within each age cohort (5–11, 12–18, and 5–18) and each MRI data modality, ensuring that normalization was strictly performed independently for each group.

Across all analyses, the same ML pipeline was applied. Feature selection and model training were performed jointly using a wrapper-based approach implemented using the Weka toolkit Witten et al. (96). Within this framework, features from the training set were first ranked using the ReliefF feature selector algorithm Kononenko (97), according to their relevance to the classification task by comparing feature values between each instance (*i.e.*, each participant) and its *k* nearest neighbors from the same and opposite classes. Based on this ranking, a series of support vector machine (SVM) models were then trained, each using a progressively reduced feature subsets, starting from the full ranked feature set and successively removing the lowest-ranked features at each iteration. Support vector machines were chosen due to their widespread use in previous neuroimaging-based autism classification studies Bahathiq et al. (98).

More precisely, *ν*-SVMs were used in this work with the radial basis function (RBF) kernel using the LibSVM library, while linear SVMs were trained using the LibLINEAR library. The kernel selection for each modality was performed empirically. More precisely, for each MRI modality and age group, both linear and RBF kernels were trained and tested, and the kernel achieving the highest cross-validated performance across the three age groups was selected as the final kernel for each modality. The linear SVM was implemented using the default hyperparameters provided by the WEKA library. For the RBF SVM, two distinct nu (*ν*) values of 0.05 and 0.04 were evaluated. This *ν* parameter regulates the trade-off between the model’s tolerance to margin errors and the proportion of support vectors. The feature subset yielding the best performance (in terms of accuracy and F1-score) was selected as the optimal subset for each model configuration. The classification methods implemented in this study have been previously used for related tasks, such as to classify other conditions (*e.g.*, depression in adults with epilepsy Delgado-García et al. (99)), or to identify risk factors associated with perinatal arterial ischemic stroke Srivastava et al. (100).

For each analysis (unimodal and multimodal) and each age group, model performance was estimated by repeatedly training and evaluating this pipeline on 30 independently generated diagnosis-balanced datasets, each maintaining a 1:1 diagnostic ratio, to mitigate the class imbalance between autistic and non-autistic participants. Therefore, for analyses conducted across the entire 5–18 years age cohort (88 autistic, 56 non-autistic), the autistic group was randomly downsampled in each iteration to match the size of the non-autistic group. In the age-stratified analyses, the 5–11 sub-group (60 autistic, 28 non-autistic) was similarly balanced by randomly downsampling the autistic group to match the non-autistic participants. The 12–18 years sub-group was already diagnosis-balanced (28 autistic, 28 non-autistic). Therefore, for consistency across analyses, 30 datasets were generated by randomly excluding one autistic and one nonautistic participant per iteration, yielding 30 balanced subsets of 27 autistic and 27 non-autistic individuals. Importantly, random undersampling was chosen over other data balancing strategies, such as synthetic oversampling techniques, to achieve more realistic performance estimates and prevent overly optimistic results due to oversampling of minority classes. Although a known limitation of undersampling methods is the potential loss of information, the repeated random undersampling across 30 iterations mitigates this concern, since different subsets of the majority class were used across iterations, ensuring that the models were exposed to the full diversity of the majority class across all data iteration runs.

For each balanced dataset, model training and evaluation were performed using a leave-one-out cross-validation (LOOCV) approach, in which a single participant was held out for testing while the remaining participants were used for training. In all cases, feature selection via ReliefF was exclusively applied to the training set at each fold, ensuring that the held-out sample never informed feature selection at any stage. Performance metrics were computed for the held-out participant in each fold and averaged across all folds and across the 30 iterations. Reported metrics included accuracy, precision, recall, F1-score, and area under the receiver operating characteristic curve (ROC-AUC). Finally, the relative contribution of the neuroimaging features to classification was assessed by computing the frequency with which each feature was selected across the 30 balanced iterations. Features that were consistently selected across iterations were interpreted as being more informative and important for the classification task, enabling the identification of neuroimaging-derived features most predictive of autism classification. Feature importance analyses were performed for the unimodal and multimodal models.

### 2.5 Statistical analyses and correlations with social responsiveness scores

To explore which MRI features influenced classification within each developmental age group (5–11 and 12–18 years), we identified the top ten features that emerged as most important for the multimodal models in each group based on their importance ranking provided by the ReliefF feature selection algorithm. This allowed us to determine whether any MRI modality had a dominant role in driving classification within each age group.

Next, we conducted statistical analyses to investigate whether these most informative features also differed significantly between autistic and non-autistic groups. For each feature, we calculated the adjusted mean value for each group and performed an analysis of covariance (ANCOVA) controlling for age and sex, allowing us to determine whether diagnostic group differences remained significant after accounting for these variables. The Bonferroni correction was used to control for multiple comparisons, and statistically significant features were identified at *p <* 0.05.

Additionally, for each narrow age group (5–11 and 12–18 years), we evaluated the association between the top ten contributing features from the multimodal models and social responsiveness scale (SRS) scores, using Pearson correlation analyses. Briefly described, the SRS is a screening questionnaire completed by parents or caregivers and is commonly used to characterize social-communication traits associated with autism. It measures multiple domains, such as social communication, awareness, cognition, motivation, and restricted or repetitive behaviors. Higher SRS scores indicate greater levels of these social-communication characteristics, with scores above 76 typically falling within the elevated range as defined by the instrument.

Three subjects with missing SRS scores were excluded from these secondary analyses: two from the 5–11 age group and one from the 12–18 age group. Bonferroni correction was applied to control for multiple comparisons and significant correlations were identified at *p <* 0.05.

## 3 Results

### 3.1 Model performance

**Table 1** summarizes the best-performing SVM classifier and kernel configuration for each neuroimaging data modality across the three age groups studied.

**Table 1:** SVM classifiers that achieved the best classification performance for each neuroimaging data modality across the three age groups studied.

Data modality	Classifier and kernel type	Additional parameters
sMRI	*ν*-SVM (LibSVM), RBF kernel	*γ* = 0.005, *ν* = 0.5
dMRI	Linear SVM (LibLINEAR), L1-regularized L2-loss (dual)	*C* = 1.0, *ϵ* = 0.001, Bias = 1.0, Tolerance = 0.1, Max. iterations = 1000
rs-fMRI	Linear SVM (LibLINEAR), L1-regularized L2-loss (dual)	*C* = 1.0, *ϵ* = 0.001, Bias = 1.0, Tolerance = 0.1, Max. iterations = 1000
Multimodal	*ν*-SVM (LibSVM), RBF kernel	*γ* = 0.005, *ν* = 0.4

**Table 2** shows the classification performance metrics obtained from the best performing SVM classifier and kernel combination specified in **Table 1** for each modeling approach, computed on the test fold of the LOOCV across the 30 data iterations. Our results show that for the unimodal ML models, the classification accuracies across the three age groups ranged between 68.3% to 75.3% for sMRI data, between 69.3% to 77.6% for dMRI data, and between 66.3% to 69.9% for rs-fMRI data. The neuroimaging data modality leading to the best classification performance was dMRI, followed by sMRI and rs-fMRI.

**Table 2:** Classification performance metrics for each modeling approach, computed on the test fold of the LOOCV across the 30 data iterations.

Age group	Modality	Accuracy	Precision	Recall	F1-score	ROC-AUC
5–18 years						
	sMRI	68.3 ± 3.3	68.4 ± 3.4	68.3 ± 3.3	68.3 ± 3.3	68.3 ± 3.3
	dMRI	70.1 ± 3.6	70.2 ± 3.7	70.1 ± 3.6	70.0 ± 3.6	70.1 ± 3.6
	rs-fMRI	66.3 ± 3.3	66.4 ± 3.3	66.4 ± 3.3	66.3 ± 3.3	66.4 ± 3.3
	Multimodal	70.5 ± 3.0	70.6 ± 3.0	70.5 ± 3.0	70.5 ± 3.0	70.5 ± 3.0
5–11 years						
	sMRI	69.0 ± 4.6	69.1 ± 4.6	69.0 ± 4.6	68.9 ± 4.6	69.0 ± 4.6
	dMRI	77.6 ± 5.5	77.7 ± 5.5	77.6 ± 5.5	77.6 ± 5.5	77.6 ± 5.5
	rs-fMRI	69.8 ± 5.5	70.0 ± 5.5	69.8 ± 5.5	69.7 ± 5.5	69.8 ± 5.5
	Multimodal	78.9 ± 5.0	79.1 ± 5.0	78.9 ± 5.0	78.9 ± 5.0	78.9 ± 5.0
12–18 years						
	sMRI	75.3 ± 4.0	75.6 ± 4.1	75.3 ± 4.0	75.3 ± 4.0	75.3 ± 4.0
	dMRI	69.3 ± 3.7	69.4 ± 3.8	69.3 ± 3.7	69.2 ± 3.7	69.3 ± 3.7
	rs-fMRI	69.9 ± 3.9	70.0 ± 4.0	69.9 ± 3.9	69.9 ± 3.8	69.9 ± 3.9
	Multimodal	76.7 ± 4.5	76.9 ± 4.5	76.7 ± 4.5	76.7 ± 4.5	76.7 ± 4.5

Overall, models that were trained on narrower age ranges achieved higher classification performance than those trained on the entire 5 to 18 years age cohort. For example, rs-fMRI models improved from an accuracy of 66.3% in the entire age cohort to 69.8% and 69.9% in the 5–11 and 12–18 age groups, respectively. A similar trend was observed with respect to the accuracies of sMRI models, which increased from 68.3% (5–18 years) to 69% (5–11 years) and 75.3% (12–18 years). Although dMRI models showed strong performance across all age groups, its highest accuracy was also obtained in the narrower 5–11 age group (77.6%). A similar trend was observed in the multimodal models, which achieved 78.9% accuracy in the 5–11 age group and 76.7% in the 12–18-year group, compared to the 70.5% accuracy of the full 5–18 age cohort. Importantly, the multimodal models outperformed the unimodal ones in all age groups.

### 3.2 Unimodal models: feature importance analyses

Next, we examined the relative contribution of structural ROI-wise volumes, diffusion ROI-wise MD and FA values, and functional ROI-wise graph-theory–based features for classification within each unimodal model for the two narrow age groups. For each modality and age group, we first computed gradient maps representing the frequency with which each region was selected across the 30 data-iteration runs. Brain regions were used as a spatial framework to visualize where features were consistently selected.

**Figure 4:**
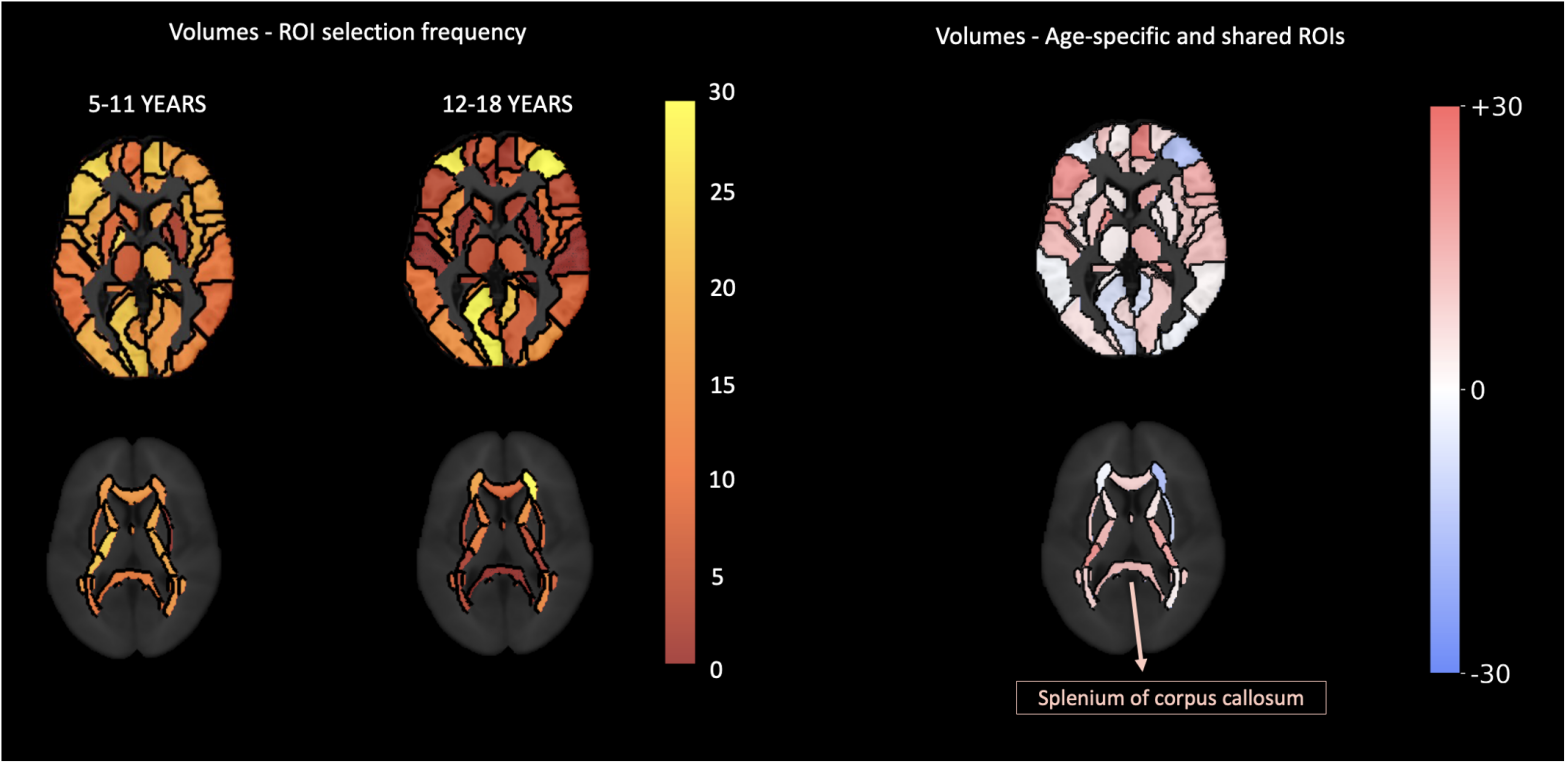
Axial slices of the AAL-90 atlas (top row) and the JHU-ICBM-DTI-48 WM atlas (bottom row), each overlaid on their respective T1-weighted templates. The left panels display gradient maps (0–30) representing the selection frequency of sMRI-derived volumetric ROIs across the 30 data-iteration runs, shown separately for the 5–11- and 12–18-year age groups. Intensities toward yellow indicate higher selection frequency. The right panels show the difference maps (Group 5–11 minus Group 12–18) for GM (top) and WM (bottom) regions, displayed on a −30 to +30 scale. Red indicates higher feature importance in the 5–11 age group, while blue indicates higher feature importance in the 12–18 age group, shown over a T1-weighted MRI anatomical background.

**Figure 5:**
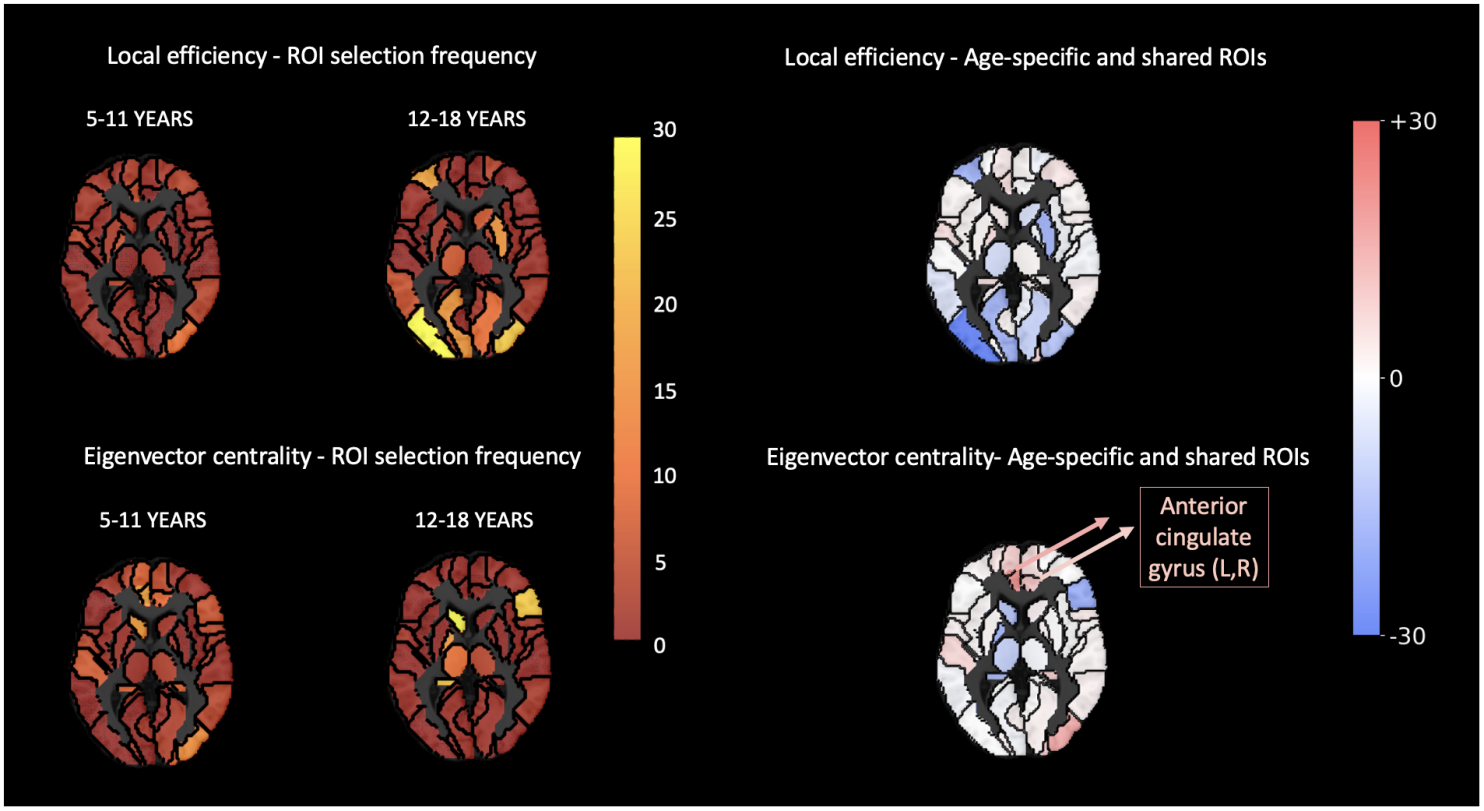
Axial slices of the AAL-90 atlas overlaid on its respective T1-weighted MRI template. The left panels display gradient maps (0–30) representing the selection frequency of ROI-wise local efficiency and eigenvector centrality features extracted from the rs-fMRI graph-theory analysis across the 30 iteration runs, shown separately for the 5–11 and 12–18-year groups. Higher intensities (toward yellow) indicate more frequent selection. The right panels show the difference maps (Group 5–11 minus Group 12–18) for local efficiency (top) and eigenvector centrality (bottom), displayed on a −30 to +30 scale. Red indicates higher feature importance in the 5–11 age group, while blue indicates higher feature importance in the 12–18 age group, shown over a T1-weighted MRI anatomical background.

**Figure 6:**
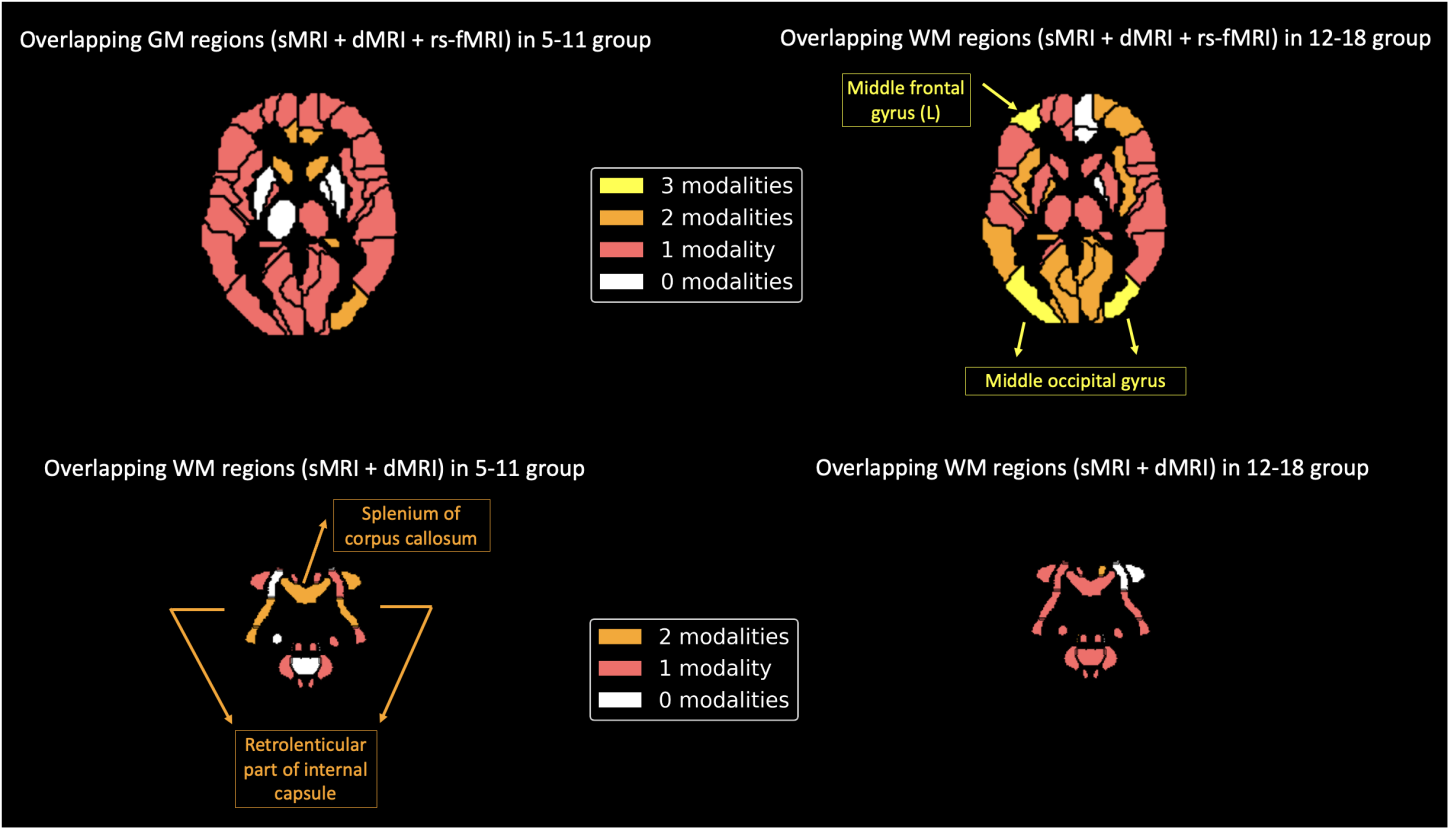
Cross-modality overlap of brain regions across MRI modalities. The top panels show the GM regions overlapping across sMRI, dMRI, and rs-fMRI, while the bottom panels show the WM regions that emerged as most discriminative across the two MRI data modalities in which WM was evaluated (sMRI and dMRI). For dMRI data, a brain region was included if it met the ≥10*/*30 threshold in at least one feature (FA or MD). For rs-fMRI data, a region was included if it met the ≥10*/*30 threshold in at least one functional feature (eigenvector centrality or local efficiency). The overlapping regions in the three MRI modalities are labeled in yellow, those overlapping in two modalities are labeled in orange, those that met the threshold in only one modality are colored in red, and those not meeting the threshold in any modality are shown in white.

We also generated difference maps (age group 5–11 minus age group 12–18) for each regional feature from each MRI data modality, displayed on a −30 to +30 scale, which enabled the identification of regions that showed higher importance in one age group relative to the other. Overall, in the sMRI models, many volumes were consistently ranked as most important for classification in both age groups, particularly in the younger group, reflected by the visible red coloration in the right panel of **Figure 4**. In contrast, the most informative regions for the dMRI models predominantly emerged from the adolescent group. However, several FA values from WM regions emerged as important in the younger age group (see **Figure 5**). Finally, in rs-fMRI models, a relatively low number of regions emerged as important in both groups, which is reflected by the red coloration in the left panel of **Figure 6**.

**Figure 7:**
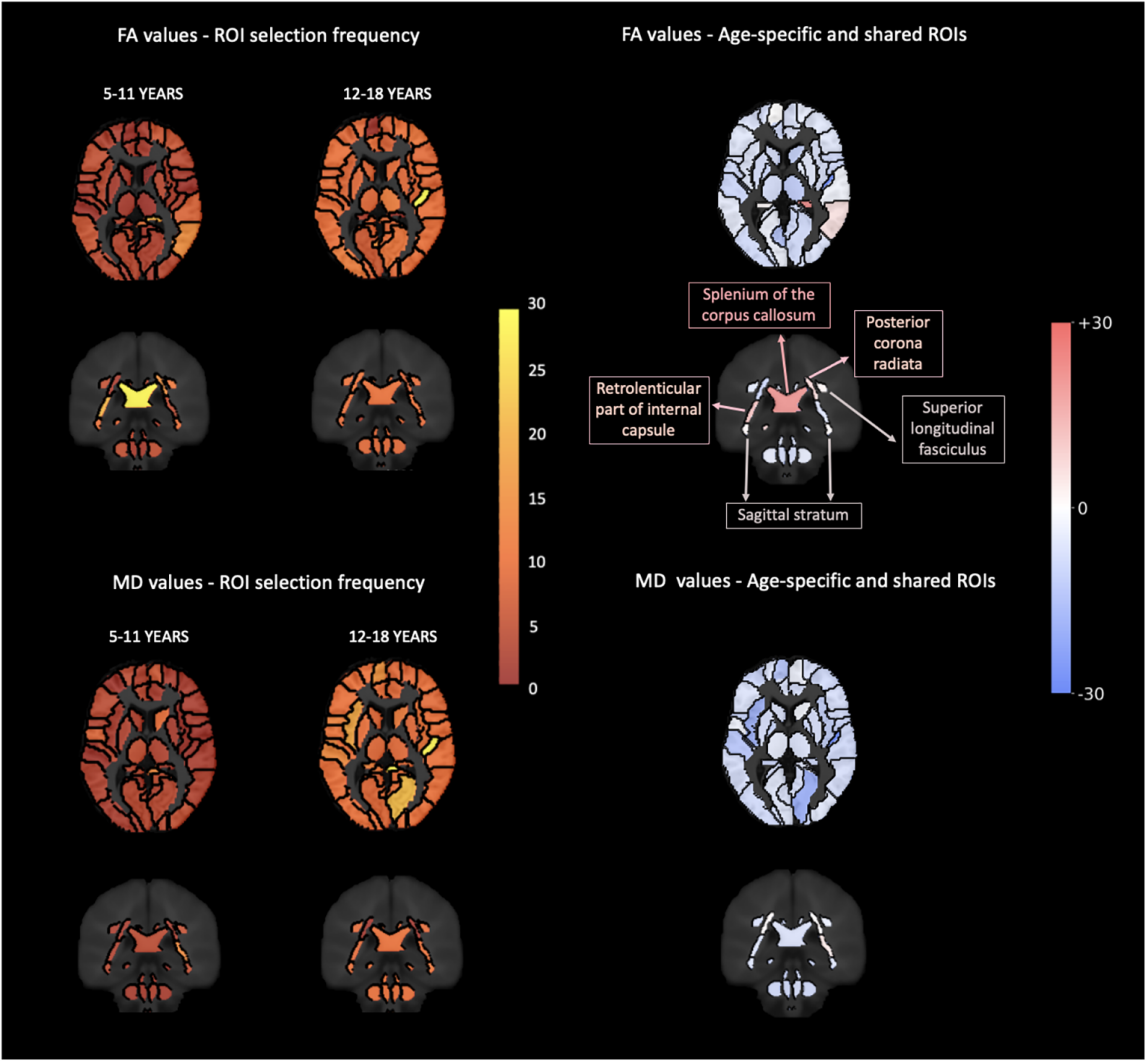
Axial slices of the AAL-90 atlas (top row) and coronal slices of the JHU-ICBM-DTI-48 WM atlas (bottom row), each overlaid on their respective T1-weighted MRI templates. The left panels display gradient maps (0–30) representing the selection frequency of ROI-wise FA and MD features extracted from the dMRI data across the 30 iteration runs, shown separately for the 5–11 and 12–18-year groups. Higher intensities (toward yellow) indicate more frequent selection. The right panels show the difference maps (Group 5–11 minus Group 12–18) for GM and WM regions for FA (top) and MD (bottom) features, displayed on a −30 to +30 scale. Red indicates higher feature importance in the 5–11 age group, while blue indicates higher importance in the 12–18 age group, shown over a T1-weighted MRI anatomical background.

Finally, we investigated whether any brain region showed consistently high predictive value across structural, diffusion, and functional features from the unimodal models, and, if so, how the importance of that region differed between the two narrow age groups. A region was considered to show meaningful predictive contribution within a modality if it was selected in at least 10 out of the 30 data-iteration runs (≥10*/*30 threshold), which corresponds to one-third of all repetitions. This threshold was selected for this analysis because it means that a variable was selected at least in one-third of all data-iteration runs, thus reducing the likelihood of selecting spurious and noise-related features. Moreover, it was found that a stricter threshold (≥15*/*30) resulted in considerably fewer regions meeting the criterion in several modalities (particularly in models trained with rs-fMRI data), leading to limited interpretability of the subsequent cross-modality overlap analysis that we performed. Thus, the ≥10*/*30 threshold was considered to be the most informative option, while still being conservative enough to discard brain features that were not consistently selected as important across many runs. To illustrate this regional cross-modality overlap, **Figure 7** shows the GM and WM brain regions that consistently emerged as most predictive across the different MRI modalities for the 5–11 and 12–18 age groups. The cross-modality overlap is visualized using a color scale: yellow indicates regions that met the ≥10*/*30 threshold in all three MRI modalities, orange indicates regions that met the threshold in at least two modalities, red indicates regions that met it in only one modality, and white indicates regions that did not meet the threshold in any modality.

### 3.3 Multimodal models: statistical analyses of the most informative features

Based on the statistical analyses between the 10 most contributing features from the multimodal models and diagnostic groups described in 3.5, **Tables 3**, **4** show the results for the younger and adolescent age groups, respectively. The ten features listed in each table are ranked from highest to lowest contribution to classification within the multimodal models. Overall, the multimodal results show that feature contributions were highly age-dependent, with dMRI features dominating in younger children, whereas adolescents showed a more multimodal distribution, with informative features more evenly being used from all three MRI data modalities. Consistent with this pattern, statistical analyses revealed that in younger children, seven dMRI-derived features showed significant differences between groups, whereas in adolescents, the significant effects were distributed across multiple MRI modalities.

**Table 3:** Statistical analyses of the top 10 most contributing features from the multimodal model in the 5–11 age group.

Region	Metric	Mean non-autistic	Mean autistic	*p*-value	Direction
**Splenium of corpus callosum**	FA	0.6789	0.6261	**0.00004**	Non-autistic *>* Autistic
**Retrolenticular part of internal capsule (L)**	FA	0.5185	0.4828	**0.0119**	Non-autistic *>* Autistic
**Inferior fronto-occipital fasciculus (R)**	FA	0.3330	0.2923	**0.0106**	Non-autistic *>* Autistic
**Hippocampus (R)**	FA	0.1354	0.1228	**0.0349**	Non-autistic *>* Autistic
Anterior corona radiata (R)	MD	0.000837	0.000859	0.1178	Non-autistic *<* Autistic
**Retrolenticular part of internal capsule (R)**	MD	0.000777	0.000809	**0.0002**	Non-autistic *<* Autistic
**Middle temporal gyrus (R)**	FA	0.1244	0.1129	**0.0286**	Non-autistic *>* Autistic
**Inferior parietal lobule (L)**	Volume	0.01453	0.01568	**0.0373**	Non-autistic *<* Autistic
Sagittal stratum (R)	FA	0.4312	0.4043	0.1222	Non-autistic *>* Autistic
Caudate (L)	Eigenvector centrality	0.0967	0.1091	0.1126	Non-autistic *<* Autistic

Statistically significant features are highlighted in bold.

**Table 4:** Statistical analyses of the top 10 most contributing features from the multimodal model in the 12–18 age group.

Region	Metric	Mean non-autistic	Mean autistic	*p*-value	Direction
**Calcarine (L)**	Volume	0.0129	0.0117	**0.0284**	Non-autistic > Autistic
**top occipital gyrus (L)**	Local efficiency	0.7507	0.8093	**0.0071**	Non-autistic < Autistic
Caudate (L)	Eigenvector centrality	0.0863	0.1008	0.1277	Non-autistic < Autistic
**Uncinate fasciculus (L)**	MD	0.000786	0.000814	**0.0331**	Non-autistic < Autistic
Thalamus (R)	Eigenvector centrality	0.0988	0.1106	0.5245	Non-autistic < Autistic
Posterior cingulum (R)	MD	0.000750	0.000771	0.0634	Non-autistic < Autistic
Putamen (L)	Local efficiency	0.7495	0.7682	1.0000	Non-autistic < Autistic
Posterior thalamic radiation (R)	MD	0.000781	0.000806	0.2421	Non-autistic < Autistic
Thalamus (L)	Eigenvector centrality	0.0997	0.1105	0.5549	Non-autistic < Autistic
**Heschl gyrus (R)**	FA	0.2064	0.1762	**0.0097**	Non-autistic > Autistic

Statistically significant features are highlighted in bold.

### 3.4 Multimodal models: association between features and social responsiveness scores

Significant correlations between multimodal model-derived features and SRS scores were identified in both age groups. **Table 5** reports only those features that showed statistically significant associations after Bonferroni correction. Visualization plots for these correlations are provided in **Supplementary Figure 1**. Briefly described, only a small subset of multimodal features showed significant associations with SRS scores. Remarkably, all significant associations were exclusively driven by dMRI-derived features. In younger children, four features showed significant correlations with SRS scores, whereas one feature reached significance in adolescents.

**Table 5:** MRI features significantly correlated with SRS scores for both the 5–11 and 12–18 age groups.

Age group	Region	Metric	r	*p*-value
5–11	Retrolenticular part of internal capsule (R)	MD	0.426	0.0005
5–11	Anterior corona radiata (R)	MD	0.352	0.0099
5–11	Splenium of corpus callosum	FA	-0.339	0.0156
5–11	Sagittal stratum (R)	FA	-0.308	0.0434
12–18	Heschl gyrus (R)	FA	-0.409	0.0213

## 4 Discussion

This study explored the potential of neuroimaging-based classification models to study autism-related neuroimaging patterns across well-defined pediatric age groups (younger participants of 5–11 years, adolescents of 12–18 years, and the entire 5–18 years age cohort). Regarding the unimodal models, the best classification results were observed when using dMRI data, followed by sMRI, and rs-fMRI. Overall, the best model classification performance results were obtained in the younger age cohort. Moreover, the multimodal classifiers outperformed the unimodal ones in all age groups. Finally, the consistency of features selected by the feature selection pipeline was explored across modalities and each narrow age group (5–11 and 12–18 years) to identify developmentally relevant neurobiological signatures of autism. The results showed that the most informative MRI-derived features varied across developmental groups.

### 4.1 Comparing the different modeling approaches

First, models trained on narrower age ranges achieved overall higher classification performance than those trained on the entire 5 to 18 years age cohort. This pattern was observed in both unimodal and multimodal models. It may be argued that that the lower performance of models trained on the entire age cohort may be linked to the developmental heterogeneity present across this wide age range.

Second, the classification accuracy across unimodal ML models ranged from 68.3% to 75.3% for sMRI, from 69.3% to 77.6% for dMRI, and from 66.3% to 69.9% for rs-fMRI data across the three age groups. Overall, ML models trained with dMRI data performed best. The models trained using rs-fMRI data achieved comparable performance to the models using the other data modalities, while requiring a relatively small number of features, suggesting that a compact set of graph theory-derived features was strong enough to capture relevant information. In line with our findings, prior work has reported that some autism-related structural changes may be stronger during early childhood and diminish or resemble more to typical development during adolescence. For example, a longitudinal study reported that whole brain volume is increased in young autistic children but decreases during adolescence, approaching typical volumes around the ages of 10 to 15 Lange et al. (30). However, in our study, the sMRI model of adolescents achieved better performance when compared to the one of younger children, indicating that during adolescence, structural features may still carry meaningful information predictive for autism classification.

Third, multimodal classifiers performed better than unimodal ones in all age groups, suggesting that they captured important information about brain structure, microstructure, and function. In the younger group (5–11 years), the multimodal model showed a small improvement in performance (78.9% accuracy) over the dMRI unimodal model (77.6% accuracy). Similarly, in the adolescent group (12–18 years), the multimodal model showed a small improvement in performance (76.7% accuracy) when compared to the sMRI unimodal model (75.3%). Performance gains were smaller when models were trained on the entire 5 to 18 years age cohort, with multimodal data integration reaching 70.5% accuracy.

Fourth, regarding the classifiers, the SVM kernel used for achieving the best classification performance varied across MRI modalities (see **Table 1**). The linear kernel led to the best results for functional and diffusion data, indicating that DTI-derived and graph theory-derived features were more linearly separable. The RBF kernel achieved the highest classification performance for structural and multimodal data, suggesting that the relationship between neuroimaging features and diagnostic labels was more complex and high-dimensional. To summarize, these results show that that the information captured in each MRI data modality can be represented and learned in a unique way, and no single SVM kernel may be optimal for all modalities.

### 4.2 Unimodal models: developmental differences in MRI feature contributions

Next, we discuss how the contribution of MRI features from unimodal models differed across developmental groups to explore potential age-related differences in predictive MRI features. First, in the models trained with sMRI-derived volumes, several brain regions consistently emerged as informative in both groups (see **Figure 4** for visualization). This matches the findings from previous literature, since multiple regional volumetric and cortical thickness alterations have been observed across childhood and adolescence in autism (*e.g.*^13^, ^14^, ^16^, ^18^, ^21^–^29^, ^31^). Notably, more regions emerged as predictive in the younger group compared to the adolescent group, reflected by the higher red-coloration in the right panel of **Figure 4**. From a neurodevelopmental perspective, this identified pattern is consistent with the hypothesis that autism-related structural brain differences are most pronounced during early childhood, with suspected accelerated brain growth and atypical cortical maturation, which tend to normalize or diverge less from typical trajectories as development progresses Lange et al. (30). Notably, the splenium of the corpus callosum emerged as being more predictive in the younger cohort compared to the adolescent group. This finding is also meaningful from a neurodevelopmental perspective because this bundle of nerve fibers undergoes substantial myelination and volume growth during childhood, which makes it particularly sensitive to atypical developmental patterns during this period Luders et al. (101). Moreover, aligned with this finding, reduced size of this region has been observed in autistic children aged 3–11 years Berkins et al. (102). Overall, multiple GM and WM regions emerging as important in both age groups may reflect a broad pattern of structural alteration across development at the whole brain level, with several brain volumes being involved, rather than the presence of alterations within a small number of volumes.

Regarding dMRI-derived FA and MD values, when compared to the adolescent group, fewer regions emerged as most predictive in the 5–11 group, these being primarily FA-derived features in WM tracts, particularly projection and association fibers, such as the corona radiata, superior longitudinal fasciculus, and sagittal stratum. Previous DTI studies in autistic children have reported microstructural alterations in these tracts when compared to typically developing peers Vogan et al. (35)Weinstein et al. (103)Barnea-Goraly et al. (104). The predominance of FA-derived features in the younger group may reflect ongoing WM myelination changes. Indeed, FA values are highly influenced by myelination processes, which are particularly active during childhood. Disruptions in myelination during childhood may also be associated with the reduced long-range cortical connectivity that has been reported in autistic children Kikuchi et al. (105), potentially affecting higher-order social cognition and executive functions. In contrast, several GM and WM regions emerged as predictive for classification for both FA and MD metrics in adolescents (reflected by the blue coloration in the right panel of **Figure 5**), suggesting that while myelination processes continue and WM matures through adolescence, FA differences may have become less extreme, while MD still captures microstructural variability. This more distributed pattern of predictive FA and MD imaging features observed in adolescents may suggests that autism-related neurobiological signatures evolve with age, further supporting the idea that autistic children and adolescents may exhibit distinct neural mechanisms that underlie different symptom expressions. Importantly, given how FA and MD values are known to change across typical development Lebel and Beaulieu (106), it was expected to observe developmental shifts in these metrics in autism. The splenium of the corpus callosum was the most important feature for classification in the younger age group, also showing relatively high importance in the adolescent group. Notably, FA decreases and MD increases have also been reported in the body, splenium, and genu of this extensively studied region both in autistic children and adolescents 35, 37–41.

With respect to rs-fMRI-derived graph-theory-based features, most regions did not consistently show high importance for classification. The few regions that emerged as important differed between age groups (see **Figure 6**), suggesting that functional phenotypes of autism may change across development, as reported in previous work. For example, connectivity differences have been found across development, with studies reporting hyper-connectivity during childhood and hypo-connectivity during adolescence Uddin et al. (47). Notably, the anterior cingulate gyrus, which is a key node of the salience network (SN), emerged among the most important in younger children. The SN is an important brain system that plays a role in detecting behaviorally relevant stimuli and integrating emotional and interoceptive information, functions that are frequently reported as atypical in autistic children Loureiro et al. (107).

### 4.3 Unimodal models: cross-modality overlap of brain regions

We investigated if any brain region showed high informativeness across MRI-derived features from the three unimodal models, and, if so, how the region differed between narrow age groups. This analysis was motivated by prior work that has reported convergent regions with structural-functional alterations in autism Mueller et al. (108), as well as spatially convergent multimodal patterns associated with autism-related social functioning Wei et al. (109). Our analysis was also grounded in the idea that a single neurobiological variation may manifest differently across distinct MRI modalities. For example, a volumetric alteration may reflect abnormal myelination (*e.g.*, reduced myelination), which could influence diffusion metrics (*e.g.*, with increased MD due to reduced tissue density). However, our results show that the regional overlap across MRI data modalities was rather limited. No GM region emerged as most important (reaching the ≥10*/*30 importance threshold) across features derived from all MRI modalities for the younger age group (see **Figure 7**). Indeed, most regions emerged as most important only in one MRI modality, predominantly sMRI. In adolescents, two regions overlapped across modalities (the left middle frontal gyrus and the bilateral middle occipital gyrus). Regarding WM regions, the splenium of the corpus callosum and the bilateral retrolenticular part of the internal capsule overlapped in the younger group across sMRI- and dMRI- derived features, whereas no WM region overlapped in the adolescent group. Overall, both in GM and WM regions, the cross-modality overlap remained limited. These findings may suggest that differences in multimodal neuroimaging features may manifest differently in each MRI data modality, being widespread and not localized in specific brain regions. Therefore, our results support the notion of conceptualizing autism as a complex and multidimensional neural condition (110–112), with spatially distinct differences in structure, microstructure, and function.

### 4.4 Multimodal models: results from statistical and correlation analyses

Results from the statistical analyses revealed several interesting findings. First, feature contributions from multimodal models were age-dependent, with dMRI features dominating in younger children and adolescents showing a more multimodal distribution. These findings do not support the presence of unique and static autism-associated neurobiological differences across development and further highlight the benefits of conducting narrow age range analyses.

Among the ten most predictive multimodal features for classification in the younger group, six diffusion-derived features (mainly FA) from key commissural, projection, and association WM regions reached statistically significant differences between autistic and non-autistic groups, including the splenium of the corpus callosum, the retrolenticular internal capsule, and the inferior frontoccipital fasciculus (see **Table 3**). All these features showed lower values in the autistic group, consistent with extensive literature reporting reductions in FA in autistic children and adolescents in important WM tracts 37–41, 103, 104, 112. In contrast, all MD features showed higher values in the autistic group, aligning with prior studies that have also observed increases in MD in WM tracts in pediatric autism Hau et al. (36) Shukla et al. (38) Alexander et al. (39)Travers et al. (41) Kim et al. (112). The observed regional FA decreases may reflect reduced fiber coherence, and/or altered microstructural organization, myelin integrity, or neuroinflammation Travers et al. (41) Lord et al. (113), while the increases in MD may be associated with disrupted myelination, increased space between fibers, or thicker axonal diameters.

Regarding the statistical analyses with SRS scores, four dMRI-derived features reached significant correlations (see **Table 5**). More precisely, two MD features exhibited positive correlations with SRS scores, while two FA features showed negative correlations. Significant associations were observed in the retrolenticular part of the internal capsule (MD, r = 0.426, p = 0.0005), anterior corona radiata (MD, r = 0.352, p = 0.0099), splenium of the corpus callosum (FA, r = -0.339, p = 0.0156), and sagittal stratum (FA, r = -0.308, p = 0.0434). Most of these brain regions are involved in social and cognitive functions and sensory processing, which suggests that there may be an association between microstructural differences and social processing in autism. These identified correlations are consistent with previous DTI studies reporting associations between WM microstructure and social/behavioral characteristics in pediatric autism (114–116). Overall, the finding that the multimodal model for the younger group identifies dMRI-derived features that significantly differ between diagnostic groups and correlate with SRS scores suggests that microstructural properties may be particularly informative during childhood, and is in line with the finding that the unimodal dMRI model achieves comparable classification performance to the multimodal one (see **Table 2**).

In contrast, in the adolescent group, the ten most predictive multimodal features were more heterogeneous. Only four of the features identified differed significantly between groups: two dMRI-derived, one sMRI-derived, and one rs-fMRI-derived features (see **Table 4**). The two dMRI-derived features were the MD of the uncinate fasciculus and the FA of the Heschl gyrus, which also showed decreased FA and increased MD in the autistic group. The middle occipital gyrus, which is a core brain region for processing of visual information, showed increased local efficiency in the autistic group, indicating that there might be a more efficient communication between this region and its neighboring regions. In line with this finding, prior studies have reported increased local connectivity (*i.e.*, connectivity between spatially proximal brain regions) in autistic adolescents in temporo-occipital regions Keown et al. (117) Maximo et al. (118). Finally, only the FA metric from the Heschl gyrus was significantly correlated with SRS scores (r = -0.409, p = 0.0213) (see **Table 5**), further suggesting that there might be an association between less organized and coherent microstructure and greater social difficulties in autism.

### 4.5 Limitations and future directions

Several limitations related to this study have to be mentioned. First, our models perform a binary classification task that does not fully capture the complexity of autism. Research has highlighted that autism is a continuum spectrum condition with multiple phenotypic expressions DiCicco-Bloom et al. (119) and neurobiological profiles Pretzsch et al. (120). Moreover, the ABIDE database may represent the less impacted autistic participants, being the ones that may more easily tolerate long MRI scanning procedures. Therefore, our results should not be interpreted as representative for all individuals across the autism spectrum. In future work, studies should move beyond the binary categorical labeling framework and explore the underlying neurobiological heterogeneity of the condition more broadly. For example, this could be achieved through implementing multimodal subtyping approaches, independent component analyses, or normative modeling methods. Second, even though the brain ROI-wise feature extraction approach implemented facilitates feature explainability analyses, it may have also obscured fine, network-level effects that could be studied in more detail using other methods, such as graph-theoretical analyses; in which brain regions could have been represented as nodes, and the structural, microstructural, and functional connections between them as edges, allowing to study the brain’s intrinsic connectivity and organization. Third, the findings of this study are constrained by the atlases used. Indeed, it is challenging to select a single parcellation atlas that is optimal to extract radiomic features for all three distinct MRI data modalities investigated in this work. As a result of using different atlases for the different modalities, the structural, microstructural, and functional properties of a given brain region may not perfectly co-localize within the same anatomical boundaries, which could contribute to the limited cross-modality regional overlap observed in this study. Fourth, even though the brain ROI-wise feature extraction approach implemented facilitates feature explainability analyses, it may have also obscured fine, network-level effects that could be studied in more detail using other methods, such as graph-theoretical analyses; in which brain regions could have been represented as nodes, and the structural, microstructural, and functional connections between them as edges, allowing to study the brain’s intrinsic connectivity and organization. Fifth, because our data inclusion criteria were restricted to participants containing data from three MRI modalities, a relatively small sample size was considered in this study. Therefore, the findings reported in this study should be considered exploratory, and additional studies incorporating larger sample sizes would be needed to reinforce their robustness. Sixth, even though the repeated random undersampling approach implemented across 30 data iterations limited information loss, this strategy may still have constrained model generalization due to the reduced training sample sizes per iteration. Finally, implementing deep learning methods to the proposed framework would be valuable to avoid the feature engineering processes conducted. This may be especially important for the processing of the complex fMRI data, for which many advanced methods have been recently proposed, which may enhance classification performance Gao et al. (121). To support these two last points, future studies would benefit from large and public databases incorporating multimodal data from more participants.

### 4.6 Conclusions

This work demonstrates the potential of multi-modal neuroimaging-based ML models complemented by feature-importance analyses for the exploration of neurobiologically relevant patterns associated with autism across distinct developmental stages. Our findings show the benefits of incorporating structural, diffusion, and resting-state functional MRI data modalities to explore the neurobiology of a complex condition like autism across multiple complementary levels. We further emphasize that developing AI models that incorporate data of children within narrow developmental age windows may be beneficial to capture condition-specific discriminative brain patterns that could potentially be less confounded by broad age-related effects. Overall, this work contributes to the application of ML-based classification methods in pediatric neurodevelopmental research.

## Data Availability

The original contributions presented in the study are included in the article/**Supplementary Material**. Further inquiries can be directed to the corresponding author.
