# Supplementary material for: Age-stratified multimodal MRI and machine learning to explore autism-related brain characteristics in youth
Source: Front Psychiatry. 2026 Jul 2;17:1841698. doi: 10.3389/fpsyt.2026.1841698 (PMC13372782; doi:10.3389/fpsyt.2026.1841698)
Supplement: Supplementary file 1 [file DataSheet1.pdf]

Supplementary Material

1 FIGURES

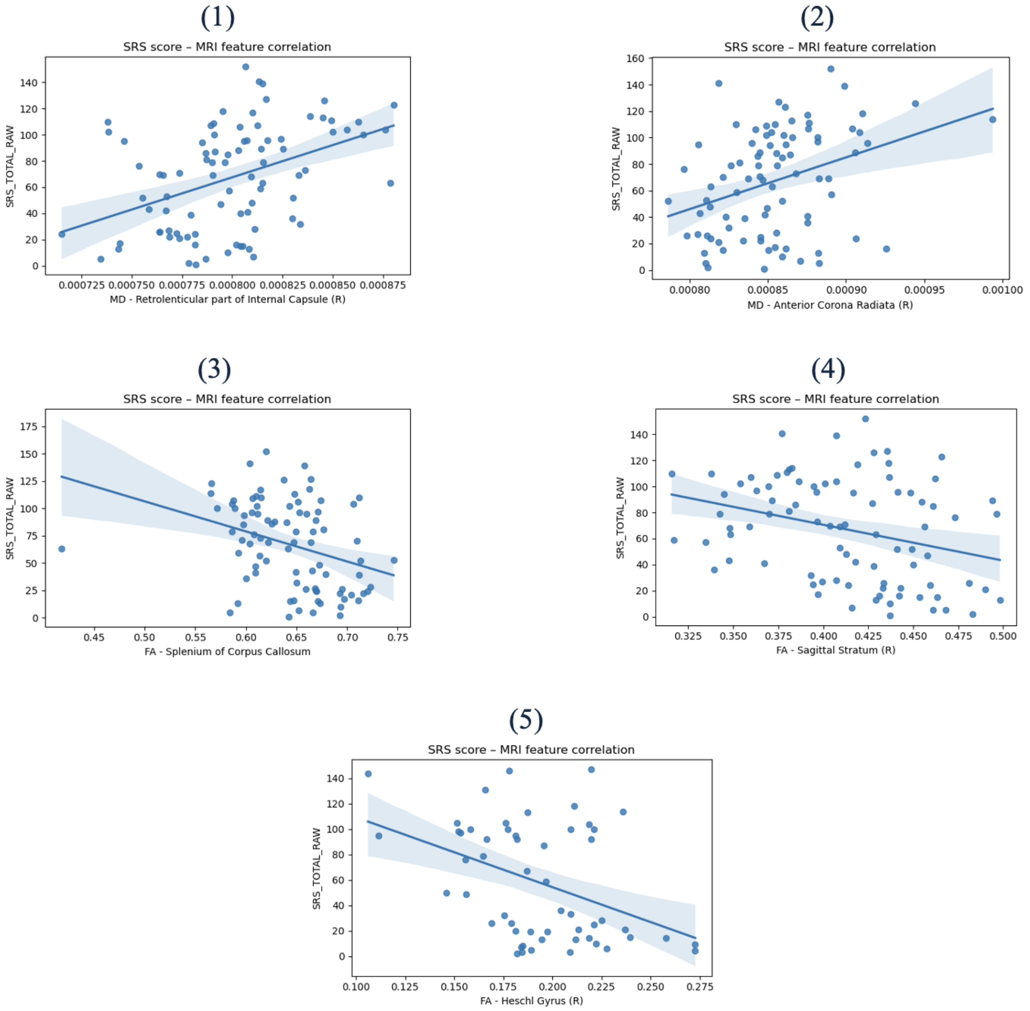

Figure S1. Scatter plots showing significant correlations between MRI features and SRS scores. Each plot displays the linear regression fit with its 95% confidence interval. These visualizations correspond to the features reported in Table 5 in the main manuscript, and include: (1) MD in the retrolenticular part of the internal capsule (R), 5-11 age group; (2) MD in the anterior corona radiata (R), 5-11 age group; (3) FA in the splenium of the corpus callosum, 5-11 age group; (4) FA in the sagittal stratum (R), 5-11 age group; and (5) FA in the heschl's gyrus (R), 12-18 age group. Higher SRS scores reflect greater severity of social impairment.

2 TABLES

Table S1. Demographic details of participants.

Diagnostic group	Autistic	Non-autistic
No. of participants	88	56
Sex (M/F)	77/11	54/2
Age (years) (mean ± SD)	10.26 ± 3.88	12.21 ± 3.49

Table S2. Number of participants in each data acquisition site.

Site	No. of participants
New York University (NYU_1 and NYU_2)	69
San Diego State University (SDSU)	53
Trinity College Dublin (TCD)	18
Barrow Neurological Institute (BNI)	4

Table S3. Distribution of autistic and non-autistic participants across ABIDE sites.

ABIDE Site	Non-autistic (n)	Autistic (n)	Total
BNI	2	2	4
SDSU	23	30	53
TCD	10	8	18
NYU	21	48	69
**Total**	**56**	**88**	**144**
